# Chromium-Based Polypyrrole/MIL-101 Nanocomposite as an Effective Sorbent for Headspace Microextraction of Methyl *tert*-Butyl Ether in Soil Samples

**DOI:** 10.3390/molecules25030644

**Published:** 2020-02-03

**Authors:** Jila Darabi, Alireza Ghiasvand

**Affiliations:** Department of Chemistry, Lorestan University, Khoramabad 6815144316, Iran; jl_kalhor@yahoo.com

**Keywords:** MIL-101(Cr), polypyrrole, headspace microextraction, methyl-*tert*-butyl ether, soil

## Abstract

The performance of headspace solid-phase microextraction (HS-SPME) was upgraded by easy and low-cost preparation of a new nanocomposite fiber. A polypyrrole/chromium-based metal–organic framework, PPy@MIL-101(Cr), nanocomposite was electrochemically synthesized and simultaneously coated on a steel wire as a microextraction sorbent. The morphology and chemical structure of the prepared nanocomposite was characterized by Fourier-transform infrared spectrometry (FT-IR), scanning electron microscopy (SEM), and energy dispersive X-ray analysis (EDX) techniques. The microsorbent was used for sampling of methyl-*tert*-butyl ether (MTBE) in solid samples, through an HS-SPME sampling strategy, followed by GC-FID measurement. The optimal experimental conditions, including extraction temperature, extraction time, and GC desorption conditions, were evaluated and optimized. The proposed procedure showed good sensitivity (limit of detection was 0.01 ng·g^−1^) and precision (relative standard deviation was 8.4% for six replicated analyses). The calibration curve was linear over the range of 5–40,000 ng·g^−1^, with a correlation coefficient of 0.994. The limit of quantification was 0.4 ng·g^−1^. The fabricated fiber exhibited good repeatability and reproducibility for the sampling of MTBE, with average recovery values of 88–114%. The intra-fiber and inter-fiber precisions were found to be 8.4% and 19%, respectively. The results demonstrated the superiority of the PPy@MIL-101(Cr)-coated fiber in comparison with handmade (polypyrrole, PPY) and commercial fibers (polyacrylate, PA; polydimethylsiloxane, PDMS; and divinylbenzene/carboxen/polydimethylsiloxane, DVB/CAR/PDMS) for the analysis of solid samples. The developed method was successfully employed for the analysis of MTBE in different soil samples contaminated by oil products.

## 1. Introduction

The idea of increasing oxygenates of full to raise and improve octane number and reduce environmental pollution has been commercialized since the 1970s [1]. The octane-booster additives, the compounds that can generate oxygen during the combustion of engine fuel, comprise organometallics, including tetraethyl lead, ferrocene and methylcyclopentadienyl manganese tricarbonyl, and organics such as methyl *tert*-butyl ether (MTBE), ethyl *tert*-butyl ether (ETBE), *tert*-amyl-methyl ether (TAME), and ethanol. The organic additives are generally less effective than organometallic octane boosters, while they are more environmentally friendly chemicals. Nowadays, MTBE is the most commonly used octane booster. It has been used in many countries since the 1990s, due to its facile manufacturing, easy mixing with hydrocarbons, increasing octane number, easy combustion, reducing emissions from engine combustion, and less corrosive than the alcoholic octane boosters. It is also used in research and industry as an alternative solvent to diethyl ether. MTBE is a volatile organic colorless liquid with a strong distinctive odor like turpentine. It has very high solubility in water (~50 g·L^−1^) at room temperature, which is 20 times higher than that of solvent gasoline. Consequently, it can easily contaminate water and soil resources with a rapid expansion [2]. Despite all benefits, there are many environmental health and safety concerns about MTBE and its possible carcinogenic properties [3]. MTBE has been banned in a few countries, but it is still used as a fuel octane booster in many developing countries. This widespread usage has resulted in a wide range of environmental pollution by MTBE. This issue will be more evident when soil contamination is taken into consideration. Natural samples like soil contain different active sites, created by organic and inorganic constituents. Organic contaminants like MTBE are usually tightly attached to these active sites, and their release from the native matrices are very difficult [4]. Therefore, development of easy, low-cost effective methods for tracing of MTBE especially in solid environmental samples is of particular importance. Several traditional and solvent-free methods for sampling and analysis of MTBE in gasoline, soil, water, and biological fluids are presented in the scientific literature, and each has its own pros and cons. These procedures usually perform in coupling with gas chromatographic (GC) instruments equipped by photo ionization detector (PID), flame ionization detector (FID), and mass spectrometry detector (MSD) [5]. On the other hand, reducing the maximum allowed amount of MTBE in water (20–40 µg·L^−1^) by the United States Environmental Protection Agency (EPA) [6] made MTBE quantification more difficult, to achieve appropriate limit of detection.

In addition to classical extraction methods, in recent years, different newly emerging techniques have been proposed for sampling, separation, and preconcentration of MTBE like luminescent bioassay [7], GC coupled with direct aqueous injection and mass spectrometry (DAI-GC/MS) [8], purge-and-trap coupled with gas chromatography (P&T/GC) [9], headspace sampling followed by GC/PID quantification [10], solid-phase microextraction (SPME), and single-drop headspace sampling coupled with GC-FID (HS-SDME–GC-FID) [11], and headspace SPME coupled with GC/MS (HS-SPME–GC/MS) [12].

DAI-GC is simpler and more operator-friendly than P&T/GC for analysis of MTBE and its degradation products [13]. However, the best alternative is HS-SPME, as its detection limit is lower than the permissible limit of MTBE in drinking water and soil, because it prevents the contact between the sample matrix and the extraction phase. Accordingly, HS-SPME can be easily applied for analysis of samples contaminated by MTBE by using available commercial fibers. However, commercial SPME fibers are expensive, fragile, have a low sorption capacity, and have limited diversity of sorbent type for sampling different analytes in various sample matrices. The selectivity of SPME for various analytes depends on the type of absorbent, to a great extent.

In recent years, various nanomaterials (NMs) have been used to improve performance of analytical methods. The selectivity and sensitivity of analytical techniques have been remarkably improved by using NMs as the sorbents [14]. High porosity, large surface area, chemical stability, physical durability, and specific affinity toward target analytes are the properties that make the NMs more beneficial than the traditional sorbent materials. These features caused NMs to attract a great attention in the analytical and bioanalytical applications of SPME [15]. In this regard, silica-based nanocomposites, different carbon-based nanosorbents, metal-organic frameworks (MOFs), and covalent organic frameworks (COFs) have been recently employed for sampling and separation of organic and inorganic analytes in different matrices [16]. MOFs or metal–organic coordination polymers (MOCPs) are microspore advanced polymeric crystalline materials that are made from hybrid inorganic–organic molecules [17]. MOFs have some unique properties, such as permanent nanoscale porosity, high surface area, costly diversity in their structures, tunable pore size, structure flexibility, and high loading capacity [18]. These compounds have been employed in various research fields, especially in separation science, gas storage, catalysis, sensor technology, drug delivery, and biomedical imaging [19]. In comparison with typical adsorbents like polyacrylate (PA), polydimethylsiloxane (PDMS), activated carbon, and zeolites (with a surface area of about 150, 450, 1500 and 300 m^2^·g^−1^, respectively), MOFs have much larger surface areas, even up to 7000 m^2^·g^−1^ [20]. Recently, the Material of Institute Lavoisier (MIL) has introduced a novel class of organic metal frameworks called MILs as super-clusters that are promising adsorbents for the analytical separation purposes [21]. MIL-101(Cr) is a porous MOF, based on chromium and terephthalate atoms, that has been used to absorb and store hydrogen gas. It has been shown that the absorption properties and the hydrothermal stability of the MOFs can be improved by preparing their composites with other materials, especially nanostructured compounds [16]. However, few MOFs nanocomposites with conductive polymers have been used as microextraction sorbents [22].

This research was aimed to develop a different type of MIL-101(Cr)/PPy nanocomposite structure on surface of a stainless-steel fiber, using an electrophoretic deposition method, for microextraction sampling purposes. The MIL-101(Cr)/PPy-coated fiber was then applied for sampling MTBE in contaminated soil samples through a HS-SPME strategy, followed by GC/FID measurement, for the first time. The results showed the superiority of the developed PPy@MIL-101(Cr)-coated fiber in comparison with PA, PPY, PDMS, and DVB/CAR/PDMS fibers for the analysis of solid samples. The developed method was successfully employed for the analysis of MTBE in different soil samples contaminated by oil products.

## 2. Results and Discussion

### 2.1. Characterization of the PPy@MIL-101(Cr) Nanocomposite Sorbent

**Figure 1:**
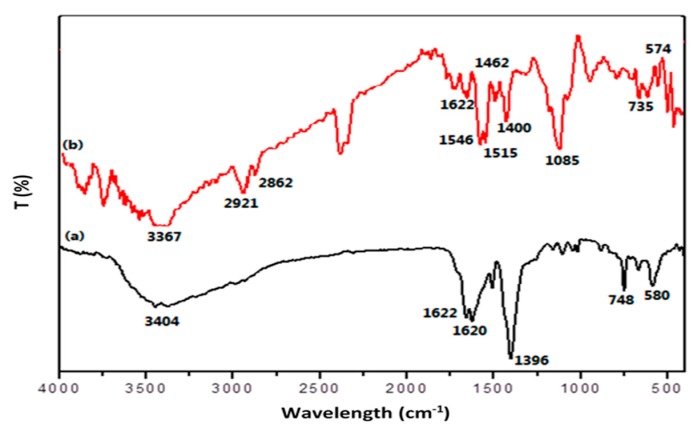
FT-IR spectra of (**a**) MIL-101(Cr) and (**b**) PPy@MIL-101(Cr) nanocomposite sorbent.

To characterize the PPy@MIL-101(Cr) structure, several batches of the nanocomposite were prepared, and their FT-IR spectra were recorded. As seen in Figure 1a, the absorption peak of 3404 cm^−1^ is attributed to –OH vibration in the water molecules that have been adsorbed or coordinated in the MIL-101 structure. The peaks appeared at 1400–1600 cm^−1^ are corresponded to the C=C stretching vibrations in the aromatic rings. The peaks in the range of 1500–1640 cm^−1^ reflects C=O stretching vibration, and 580 cm^−1^ is related to Cr–O vibration. The absorption bands at 1650 and 1620 cm^−1^ are attributed to O–C–O asymmetric vibrations. From Figure 1b, bands at 1220, 1160, 1085, and 1042 can be attributed to C–C–C and C–N–H bending, as well as C–N stretch and N–H vibration, in aromatic ring of polypyrrole. The bands at 1500–1650 cm^−1^, corresponding to the C=C vibration of the benzene ring, are like those of the MIL-101(Cr) structure. It must be noted that there are remarkable red shifts in the absorption bands in the PPy@MIL-101(Cr) structure. This fact demonstrates that the PPy@MIL-101(Cr) nanocomposite has been formed in the doping mode. Appearing stretched vibrations of C–N at 1462 cm^−1^ is another confirmation for doping PPy in the MIL structure.

**Figure 2:**
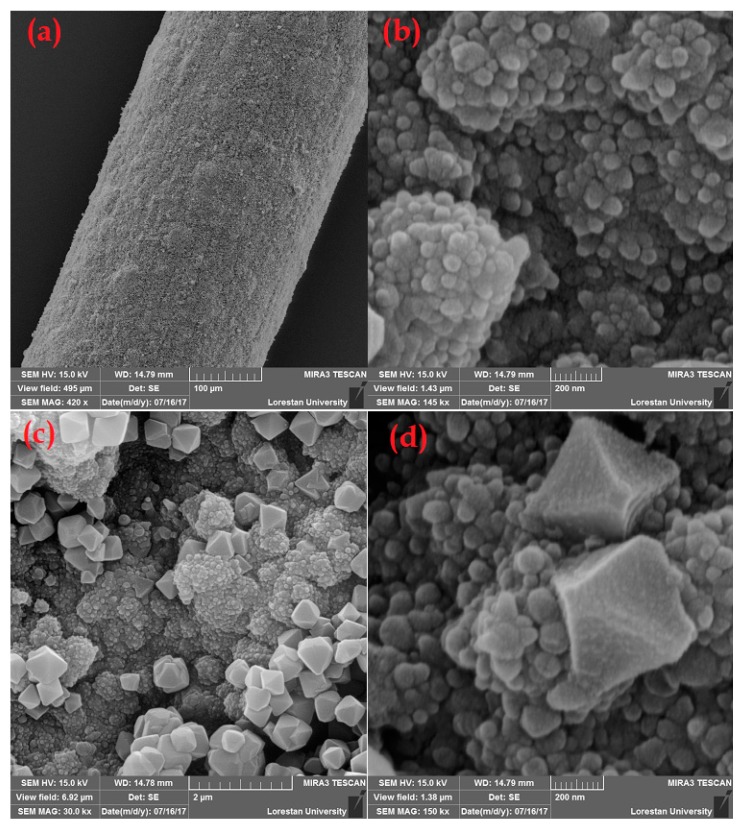
SEM images of the PPy@MIL-101(Cr) nanocomposite at different magnifications; (**a**)100 μm, (**b**,**d**) 200 nm, and (**c**) 2 μm.

The sorbent morphology was studied by using SEM, as is shown in Figure 2. The apparent uniformity and smoothness of the coating is evident in Figure 2a, while it was resistant to scratching and damage. The remarkable porosity of the nanocomposite sorbent can be seen in Figure 2b,c. The octahedral configuration of the MIL-101(Cr) molecules, grafted inside the PPy layers, is clearly obvious in Figure 2d. Additionally, penetration of the PPy particles into the MIL-101(Cr) cavities is visible on the SEM images. Furthermore, the porosity, large surface area, durability, and physicochemical stability of the nanocomposite was demonstrated during the extraction and desorption (at high temperature) experiments. The positive effect of the roughness of the fiber substrate on the durability and physical strength of the coating was also proved. Its effectiveness was comparable with the platinization, while it is a simpler, faster, lower-cost process [23]. Homogeneous distribution of the nanocomposite elemental constituents was ascertained by using the EDX mapping experiments (the results are not shown).

### 2.2. Optimization of the Extraction Procedure

To optimize the headspace sampling of MTBE in soil samples, using the PPy@MIL-101(Cr) coated SPME fiber, and obtain the best extraction and determination conditions, different affecting experimental variables were evaluated and optimized. Then, the proposed nanocomposite fiber was compared with PPy handmade fiber, as well as PA, PDMS, and divinylbenzene/carboxen/polydimethylsiloxane (DVB/CAR/PDMS) DVB/CAR/PDMS commercial fibers, under the optimal conditions.

#### 2.2.1. Extraction Time

**Figure 3:**
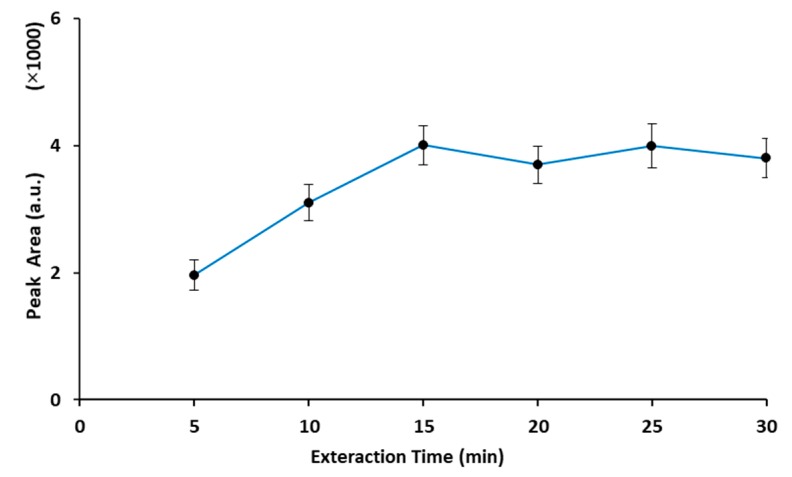
Effect of extraction time on the extraction efficiency of the HS-SPME–GC method for analysis of MTBE in solid samples. (Analyte concentration: 2 μg·g^−1^, extraction temperature: 30 °C, desorption condition: 2 min at 250 °C.)

The HS-SPME extraction is an equilibrium process, and the extraction time is the major affecting variable on the distribution coefficient of the analyte between the sample/headspace and the headspace/fiber. Therefore, to obtain the minimum time required to set up an equilibrium between the headspace and the fiber coating, the extraction time was investigated in the range of 5–30 min (Figure 3). According to the results, the amount of extracted analyte increased by increasing of extraction time up to 15 min, and then remained almost constant. Hence, 15 min was selected as the optimum extraction time for further studies.

#### 2.2.2. Extraction Temperature

**Figure 4:**
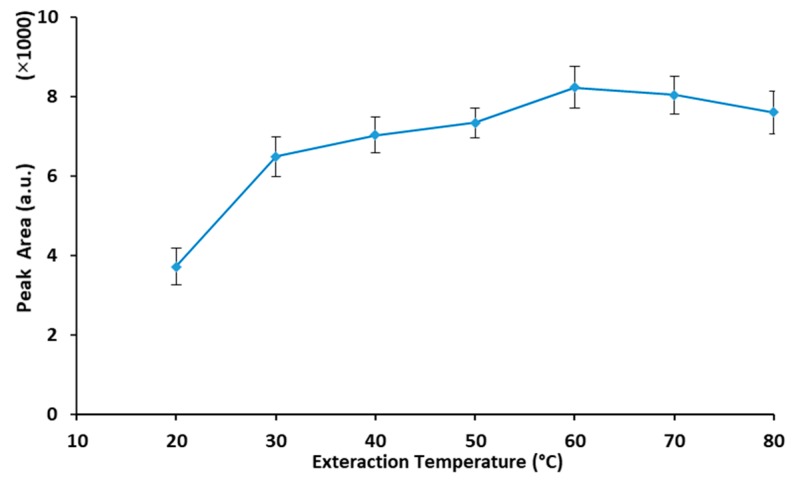
Extraction temperature vs. extraction efficiency of MTBE (analyte concentration: 2 μg·g^−1^, extraction time: 15 min, desorption condition: 2 min at 250 °C).

Effect of temperature on the extraction efficiency of HS-SPME sampling method has been well demonstrated. Rising sample temperature helps analytes to be released from the matrix and speeds up their diffusion into the headspace. Consequently, the analyte distribution coefficient (headspace/sample) increases at high temperatures. On the contrary, the distribution coefficient of analyte between fiber coating/headspace decreases. Therefore, a balance point between these two opposite effects should be obtained by optimizing extraction temperature. In this study, the effect of sample temperature was studied over the range of 20–90 °C, while other conditions were kept constant. The average peak areas (for three replicated experiment) vs. extraction temperature is depicted in Figure 4. As seen, with rising extraction temperature up to 60 °C, peak areas increase and, after that, start to decline slightly. Therefore, 60 °C was selected as the best temperature for the HS-SPME sampling of MTBE in solid samples.

#### 2.2.3. Desorption Condition

**Figure 5:**
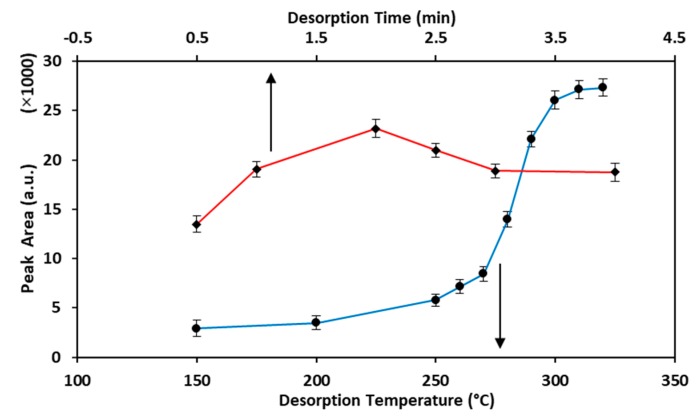
Study of desorption time and desorption temperature of the HS-SPME–GC-FID procedure for analysis of MTBE in solid samples (analyte concentration: 2 μg·g^−1^, extraction time: 15 min, extraction temperature: 60 °C).

Desorption time and desorption temperature are two important variables that affect the accuracy and precision of the HS-SPME–GC analysis, especially when using a handmade fiber. Therefore, the effect of desorption condition was investigated by applying 0.5, 1, 2, 2.5, 3, and 4 min as the desorption time, and 150, 200, 250, 260, 270, 280, 290, 300, 310, and 320 °C as the desorption temperature. The results are represented in Figure 5. In accordance with the results, the best time for complete desorption of the analyte from the fiber was 2 min, while 300 °C was obtained as the best desorption temperature. This desorption temperature seems a bit high for MTBE as a volatile molecule (boiling point: 55.2 °C). It can be due to the deep penetration of analyte into the sorbent pores and cavities with stronger interactions, which require higher temperature and longer times to be desorbed.

### 2.3. Comparison of the PPy@MIL-101(Cr)-Coated Fiber with Commercial Fibers

**Figure 6:**
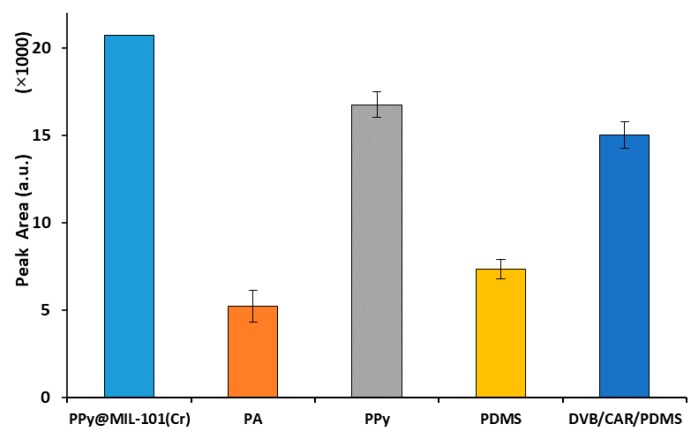
Comparison of the PPy@MIL-101(Cr)-coated SPME fiber with commercial fibers and PPy handmade fiber for sampling of MTBE in solid samples.

Physicochemical characteristics of fiber are the most important criteria in SPME sampling, as a well-known fact. Accordingly, many SPME development studies are concerned with the preparation of new fiber coatings for wider applications diversity and more effective and selective trapping of analytes of interest. In this research, the PPy@MIL-101(Cr) coated fiber was prepared to achieve more efficient extraction of MTBE as a semi-polar compound (with relative polarity of 0.124 and eluant strength of 0.20. It was anticipated that the PPy@MIL-101(Cr) fiber would have higher extraction performance due to the larger sorptive surface area and more strong interactions with the analyte, in comparison with the commercial fibers. On the other hand, incorporation of MIL-101(Cr) with PPy into a single sorbent was expected to show much higher extraction efficiency, because of significant adsorption features of MILs. Therefore, to assess these hypotheses and to evaluate the reliability of the prepared PPy@MIL-101(Cr) fiber, it was compared with PPy handmade fiber, as well as with PA, PDMS, and DVB/CAR/PDMS commercial SPME fibers. As is clear in Figure 6, under the same conditions, the PPy@MIL-101(Cr)-coated fiber showed higher extraction efficiency for the target analyte, in comparison with all examined fibers. It is noticeable that the amount of MTBE extracted by the new fiber was 64% higher than PDMS, as a general fiber for volatile and semi-volatile compounds. It was also much higher than that of DVB/CAR/PDMS commercial fiber (28%), while DVB/CAR/PDMS has been distinguished as a very suitable sorbent for many non-polar and semi-polar analytes in fiber [24].

### 2.4. Analytical Performances

Analytical performance of the proposed HS-SPME–GC-FID strategy was assessed by obtaining analytical figures of merit, including linear dynamic ranges (LDR), limit of detection (LOD), limit of quantification (LOQ), and relative standard deviation (RSD). Calibration curve was linear over the range of 5–40,000 ng·g^−1^, with a correlation coefficient of 0.994. LOD and LOQ were obtained as 0.01 and 0.4 ng·g^−1^, respectively. To evaluate the repeatability of the method, RSD was calculated based on six repeated analyses of 2 μg·g^−1^ of the analyte. It was found to be 8.4%. Inter-day RSD (reproducibility) was calculated 19% by repeating an experiment in six different days, at the same conditions. The inter-fiber reproducibility was also obtained (26%). The big intra-fiber RSD is probably due to the nature of electropolymerization process, with different affecting variables.

For further demonstrating the superiority of the developed HS-SPME–GC-FID method with PPy@MIL-101(Cr) nanosorbent, its analytical performances were compared with some similar procedures that are reported in the literature [25,26,27,28,29,30,31,32]. The results are summarized in Table 1. As is clear from the results, the HS-SPME–GC analysis using PPy@MIL-101(Cr)-coated fiber has wider linear range than all other cited methods. Its LOD and RSD are bigger or comparable with the mentioned procedures. However, it should be considered that this strategy is capable for direct analysis of complicated solid samples, while other methods have been applied in water media and some of them have used MS as much sensitive detection system.

### 2.5. Determination of MTBE in Contaminated Soil Samples

For further elucidation of applicability of the PPy@MIL-101(Cr) coated fiber, it was evaluated for HS-SPME sampling of MTBE in contaminated soil samples, followed by GC-FID quantification. For this purpose, six soil samples were collected from the area of an oil-refining company and some gas stations in different locations of Kermanshah City (Kermanshah, Iran) and subjected to the developed method. When volatile compounds are to be determined, sampling is critical, and precautions should be taken to prevent loss of analytes. Therefore, sample is needed to be analyzed as soon as possible after sampling. Otherwise the sample should be stored in a clean and tightly closed container and transported to the laboratory under cool conditions. In this research, the soil samples were collected according to international standard method of ISO 10381-1 [33], and then they were immediately transferred to the laboratory, in a closed polyethylene container, and stored in a refrigerator, at −18 °C. On the test time, the sample containers were removed from the refrigerator and allowed to reach to ambient temperature, and then they were analyzed by using the developed method, without any pretreatment step. For more assurance of the validity of the obtained data, each sample was also fortified with a known amount of MTBE and analyzed three times. The results are shown in Table 2.

## 3. Experimental

### 3.1. Materials

Analytical reagent-grade MTBE (>97%) was kindly provided by Kermanshah Oil Refining Company. It was re-distilled by using a rotary evaporator, prior to use and stored in a fridge. A stock solution (2000 µg mL^−1^) was prepared by dissolving the appropriate amount of MTBE in double-distilled water and stored at 4 °C. The working standard solutions were prepared daily by appropriate dilution of the stock solution in double-distilled water. Due to the high volatility and to minimize the MTBE evaporation, during the solution making, the distilled water was first chilled to lower than 10 °C, and then MTBE was added to it. The vial was caped, shaken up, and allowed to reach ambient temperature and then made up to the mark with distilled water in the volumetric flask. Hexaaquachromium (III) nitrate trihydrate ([Cr(H_2_O)_6_](NO_3_)_3_.3H_2_O, 98%) and terephthalic acid (1,4-benzene dicarboxylic acid, H_2_BDC, 98%) were bought form Sigma Aldrich (Steinheim, Germany). Pyrrole (>99%) was provided by Fluka (Bucks, Switzerland), stored in the fried in the dark, and re-distilled prior to each use. Lithium perchlorate (LiClO_4_·3H_2_O, 99%), *N*,*N*-dimethylformamide (DMF), methanol, and nitric acid were also purchased from Merck (Darmstadt, Germany).

### 3.2. Instruments and Conditions

A GC-2010 Plus AF gas chromatograph (Shimadzu, Kyoto, Japan), equipped with a split/splitless injector (SPL-2010 Plus), a BP-5 capillary column (30 m × 0.25 mm × 0.25 µm), a flame ionization detector (FID-2010 Plus), and GC solution software (version 2.4), was applied for chromatographic determination. Nitrogen (99.999%) was used as the carrier gas, with a constant flow rate of 1 mL min^−1^. The GC injector was run in the split mode, with a split ratio of 1:10. The GC-FID temperature program started at 35 °C, held 5 min. Then, temperature increased to 250 °C, at a rate of 50 °C min^−1^, with a hold time of 2 min, so the total time of chromatographic run was 11.3 min. The FID and injector were both maintained at 300 °C during the analysis. Zero-air and hydrogen were applied as the FID gases, and nitrogen as the make-up gas, at flow rates of 300, 30, and 25 mL·min^−1^, respectively. A VEGA-TESCAN (TESCAN, Brno, Czech Republic) field-emission scanning electron microscope (FE-SEM) was used to investigate the surface morphological features of the nanocomposite. Fourier transform infrared spectra were recorded by using a Shimadzu FT-IR 8400 spectrometer (Kyoto, Japan). It was used to characterize the functional groups of the nanocomposite. Stainless-steel wires (2.5 cm pieces) with a diameter of 0.06 mm were used as the SPME fiber. Polyacrylate (PA), polydimethylsiloxane (PDMS), and divinylbenzene/carboxen/polydimethylsiloxane (DVB/CAR/PDMS) SPME commercial fibers were purchased from Supelco (Bellefonte, PA, USA) and preconditioned according to the manufacturer’s recommended procedure. A PS-302D DC-power supply (Dazheng, Shenzhen, China) was used for the electrochemical deposition of the sorbent on the fiber. A Teflon-lined autoclave bomb was used for hydrothermal synthesis of MIL-101(Cr).

### 3.3. Preparation of MIL-101(Cr)

MIL-101(Cr) was synthesized according to an amended version of the hydrothermal method that was proposed by Frey et al. [34]. For this purpose, chromium nitrate (800 mg), terephthalic acid (332 mg), and water (9.5 mL) were blended and sonicated for a short time, resulting in a dark-blue-colored suspension. The suspension was transferred into the autoclave bomb and kept in an oven at 220 °C, for 8 h. After cooling the mixture at room temperature, a green precipitate appeared. To remove terephthalic acid residual from the nanoporous framework cavities and pores, the precipitate was refluxed with 50 mL of DMF (60 °C), for 3 h, and 50 mL of ethanol (60 °C), for 2 h, three times. To ensure complete removal of the solvents, after each reflux, the precipitate was centrifuged at 9000 rpm for 5 min. Finally, the resulting solid was dried at room temperature, overnight, and stored in a glass container. About 90% of the interior space of MIL-101(Cr) was demonstrated to be empty, after removal of the solvents. For that reason, its apparent specific surface area is very large, and that is why it turns into a strong absorbent.

### 3.4. Fabrication of the SPME Fiber Coated by PPy@ MIL-101(Cr)

The stainless-steel wire was roughened by using a coarse sandpaper and washed with ethanol and distilled water. Then, it was immersed into a 1 M nitric acid solution for 60 s, followed by rinsing with water and ethanol. Then, prepared stainless-steel wire was electrochemically coated with PPy@MIL-101(Cr) nanocomposite, using a two-electrodes electrochemical system. MIL-101 is a good preservative for polymers, the combination of which can be a new nanocomposite that produces properties of both materials, including the high thermal resistance, sorption capability, high electrical conductivity, etc. For this purpose, 0.03 g of MIL-101(Cr), 0.532 g of lithium perchlorate, 1.442 g of SDS, and 0.68 g of sodium dihydrogen phosphate were added to 40 mL of water in a 50 mL volumetric flask and homogenized for 1 h, using the ultrasonic bath. After cooling to room temperature, 350 μL of freshly distilled pyrrole was added, and the flask was capped and sonicated for 10 min. After setting the temperature of the mixture at 20 °C, the flask was made up to the mark with water and mixed well. A 5 mL portion of the mixture was poured into the electrochemical cell, and the prepared stainless-steel wire was applied as the anode. A platinum wire was used as the cathode and a constant DC voltage applied. The mixture was continuously stirred during this period (750 rpm). In this way, pyrrole is oxidized, transported, and electropolymerized on the anode surface, while simultaneously, MIL-101(Cr) molecules are also transported and enfolded into the polypyrrole layers on the stainless-steel fiber. After 50 min, the DC power supply voltage was turned off, and the PPy@MIL-101(Cr)-coated steel fiber was removed and conditioned in an oven, at 300 °C, for 30 min. Finally, the fiber was mounted on a handmade fiber holder and carried out for the headspace microextraction experiments. Comparison of different coatings in terms of electrolyte type, coating time, coating voltage, adhesion, and shape of the electrodeposited layers showed that LiClO_4_ was the most suitable electrolyte, which produced more uniform and sticky coatings. Thus, to carry on the work, 0.532 g of LiClO_4_ was used, with a constant voltage of 1.4 V, for 50 min. Brunauer–Emmett–Teller (BET) analysis of the prepared nanocomposite showed that reduced surface areas (*S*BET) for MIL-101(Cr) and PPy@MIL-101(Cr) were about 3500 and 1600 m^2^·g^−1^, respectively, which demonstrated that the pyrrole molecules occupy the pores inside the MIL-101(Cr) structure.

### 3.5. HS-SPME Procedure

The PPy@MIL-101(Cr)-coated stainless-steel fiber was used for the sampling of MTBE in soil, followed GC-FID quantitative measurement. For this purpose, a standard sand sample was used as the model matrix. The sand composition is very similar to typical soils and therefore can be used as a model matrix for soil studies. In a 10 mL SPME vial, 20 μL of MTBE solution (200 ppm) was added to 2.0 g standard sand, to achieve a mass concentration of 2 μg g^−1^. The vial was capped and shaken manually for 1 min for homogeneous mixing of the standard solution and sand. For complete adsorption and equilibration of MTBE with the sand matrix, the vial was left at room temperature overnight. For HS-SPME–GC-FID analysis, the vial was transferred to the water bath (60 ± 1 °C), and the SPME fiber was exposed to the headspace of the sample for 15 min, for trapping of MTBE and equilibration between the fiber coating and the sample headspace. After that, the fiber was retracted and immediately injected into the injection port of the GC system, for thermal desorption of the extracted MTBE (at 300 °C for 2 min).

## 4. Conclusions

MILs are superior to other MOFs for microextraction purposes due to their unique features like high surface-to-volume ratio, porosity, lower cost, and excellent chemical/mechanical stability. In this research, MIL-101(Cr) was enfolded into PPy layers during the electropolymerization of pyrrole on the surface of a stainless-steel wire. The coated fiber was applied as a novel and efficient sorbent for HS-SPME–GC sampling and quantification of MTBE in solid samples, without any sample pretreatment step. A comparison of figures of merit of PPy@MIL-101(Cr) nanocomposite with commercial SPME fibers demonstrated its superiority for precise and accurate determination of MTBE in contaminated soil samples. The proposed HS-SPME–GC method is solvent-free, fast, and simple in comparison with international standards. In this procedure, MTBE content of contaminated soil samples can be analyzed directly, without further sample preparation, and so there is no possibility of analyte loss. For filed analysis, complicated soil samples can be collected in SPME vials, transferred to laboratory (without considering and adjusting of the environmental parameters), and directly subjected to GC analysis. Unlike other methods, there are no limitations in terms of sample storage conditions and time. The developed procedure showed acceptable analytical performances compared to some of the most important similar methods that are reported in the literature. It was successfully applied for measurement of MTBE in contaminated soil samples.

## Figures and Tables

**Table 1:** Comparison of the developed HS-SPME–GC-FID method with some of the important reported procedures, for the extraction and determination of MTBE.

Method	Fiber	RSD%	LOD	LDR	Sample	Ref.
HS-SPME–GC-FID	IL-mediated PDMS-MWCNTs	6.5	0.007 ng·mL^−1^	0.03–200 ng·mL^−1^	Water	[25]
HS-SPME–GC–MS	DVB/CAR/PDMS	<10	0.03 μg·L^−1^	0.10–40 μg·L^−1^	Blood serum	[26]
HS-SPME–GC-FID	DVB/CAR/PDMS	6–8	0.02 μg·L^−1^	0.1–400 μg·L^−1^	Water	[27]
HS-SPME-IMS	PDMS/CAR	8.3	5 mg·L^−1^	10–1390 ng·L^−1^	Water	[28]
HS-SPME–GC–MS	Carboxen-PDMS	10–11	10 ng·L^−1^	5–250 ng·L^−1^	Water	[29]
HS-SPME–GC–MS	SWCNTs	<15	10 ng·L^−1^	100–5000 ng·L^−1^	Urine	[30]
HS-SPME–GC–MS	Carboxen-PDMS	5	1.5 ng·L^−1^	0.3–2.4 ng·L^−1^	Whole blood	[31]
HS-SPME–GC–FID	PDMS/DVB	6.3	0.45 μg·L^−1^	5–500 μg·L^−1^	Water	[32]
HS-SPME–GC-FID	PPy@MIL-101(Cr)	8.4	0.01 ng·g^−1^	5–40,000 ng·g^−1^	Soil	This work

**Table 2:** Analysis and recovery of MTBE in polluted soil samples, using the HS-SPME–GC-FID procedure.

Soil Sample	Added (µg ·g^−1^)	Determined ± SD ^a^ (µg·g^−1^)	Recovery (%)
Kermanshah Oil Refinery Company (sampling from the surface of soil)	0	1.16 ± 0.51	-
	2	3.30 ± 0.08	107
Kermanshah Oil Refinery Company (sampling from the depth of soil)	0	1.54 ± 0.25	-
	2	3.61 ± 0.17	103
Kabir gas station (sampling from surface soil behind the official building)	0	1.41 ± 0.25	-
	0.5	1.98 ± 0.17	117
Besat gas station (sampling from surface soil of gas station area)	0	0.92 ± 0.48	-
	1	1.89 ± 0.05	96
Shahrvand gas station (sampling from surface soil of gas station area)	0	2.09 ± 0.34	-
	2	4.03 ± 0.09	97
Shahrvand gas station (sampling from firefighting soil bucket)	0	2.72 ± 0.50	-
	2	4.48 ± 0.08	88

**^a^** standard deviation for three replicated measurements.
